# Dichloridobis(1-ethyl-2,6-dimethyl­pyridinium-4-olate-κ*O*)zinc(II)

**DOI:** 10.1107/S160053681004119X

**Published:** 2010-10-23

**Authors:** M. Thenmozhi, A. Philominal, S. Dhanuskodi, M. N. Ponnuswamy

**Affiliations:** a Centre of Advanced Study in Crystallography and Biophysics, University of Madras, Guindy Campus, Chennai 600025, India; b Department of Physics, Bharathidasan University, Tiruchirappalli 620024, India.

## Abstract

In the title compound, [ZnCl_2_(C_9_H_13_NO)_2_], the Zn^II^ ion is coordinated by two Cl^−^ anions and two O atoms of two zwitterionic organic ligands in a distorted tetra­hedral arrangement. In the crystal, mol­ecules are linked into sheets parallel to the *bc* plane by C—H⋯Cl and C—H⋯O hydrogen bonds and weak π–π inter­actions [centroid–centroid distance = 3.669 (1) Å].

## Related literature

For general background to pyridinium compounds, see: Anwar *et al.* (1997 ▶, 1999 ▶); Damiano *et al.* (2007 ▶); Darensbourg *et al.* (2003 ▶); Mootz & Wusson (1981 ▶). For hydrogen-bond motifs, see: Bernstein *et al.* (1995 ▶). For the preparation of 1-ethyl-2,6-dimethyl-4(1*H*)-pyridinone trihydrate, see: Garratt (1963 ▶).
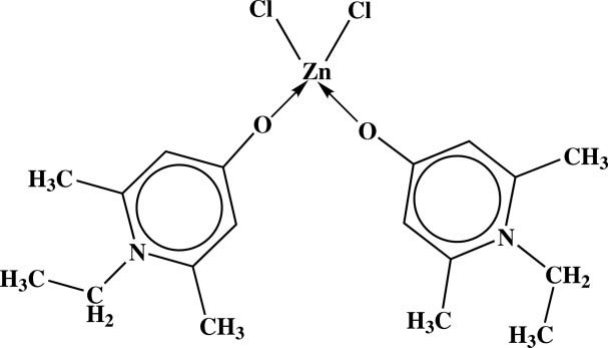


## Experimental

### 

#### Crystal data

[ZnCl_2_(C_9_H_13_NO)_2_]
*M* *_r_* = 438.68
Monoclinic, 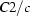

*a* = 30.365 (2) Å
*b* = 8.5366 (6) Å
*c* = 15.7982 (12) Å
β = 94.281 (4)°
*V* = 4083.7 (5) Å^3^
*Z* = 8
Mo *K*α radiation
μ = 1.48 mm^−1^
*T* = 293 K
0.25 × 0.25 × 0.23 mm

#### Data collection

Bruker SMART APEXII area-detector diffractometer
Absorption correction: multi-scan (*SADABS*, Bruker, 2008 ▶) *T*_min_ = 0.709, *T*_max_ = 0.727
19140 measured reflections
5069 independent reflections
4248 reflections with *I* > 2σ(*I*)
*R*_int_ = 0.042

#### Refinement

*R*[*F* ^2^ > 2σ(*F* ^2^)] = 0.031
*wR*(*F* ^2^) = 0.084
*S* = 0.99
5069 reflections
233 parameters
H-atom parameters constrained
Δρ_max_ = 0.61 e Å^−3^
Δρ_min_ = −0.56 e Å^−3^

### 

Data collection: *APEX2* (Bruker, 2008 ▶); cell refinement: *SAINT* (Bruker, 2008 ▶); data reduction: *SAINT*; program(s) used to solve structure: *SHELXS97* (Sheldrick, 2008 ▶); program(s) used to refine structure: *SHELXL97* (Sheldrick, 2008 ▶); molecular graphics: *ORTEP-3* (Farrugia, 1997 ▶); software used to prepare material for publication: *SHELXL97*  and *PLATON* (Spek, 2009 ▶).

## Supplementary Material

Crystal structure: contains datablocks global, I. DOI: 10.1107/S160053681004119X/ci5172sup1.cif

Structure factors: contains datablocks I. DOI: 10.1107/S160053681004119X/ci5172Isup2.hkl

Additional supplementary materials:  crystallographic information; 3D view; checkCIF report

## Figures and Tables

**Table 1:** Selected bond lengths (Å)

Cl1—Zn1	2.2292 (5)
Cl2—Zn1	2.2349 (6)
O1—Zn1	1.9649 (13)
O2—Zn1	1.9472 (13)

**Table 2:** Hydrogen-bond geometry (Å, °)

*D*—H⋯*A*	*D*—H	H⋯*A*	*D*⋯*A*	*D*—H⋯*A*
C8—H8*A*⋯O1^i^	0.96	2.53	3.344 (3)	143
C10—H10*A*⋯Cl1^ii^	0.96	2.82	3.740 (2)	162
C10—H10*C*⋯Cl1^iii^	0.96	2.82	3.745 (2)	162
C13—H13⋯Cl2^iv^	0.93	2.82	3.709 (2)	161

Symmetry codes: (i) 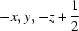; (ii) 
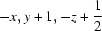
; (iii) 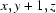; (iv) 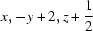.

**Fig. 1.**
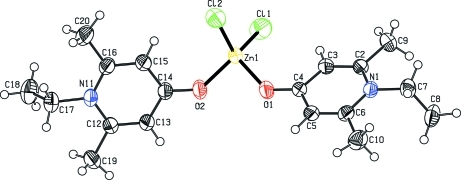
The molecular structure of the title compound, showing 50% probability displacement ellipsoids.

**Fig. 2.**
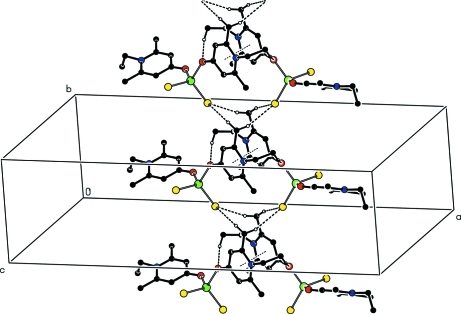
The crystal packing of the title compound. H atoms not involved in hydrogen bonding (dashed lines) have been omitted for clarity.

## supplementary crystallographic information

### Comment

Organic pyridinium salts have been widely used as guest molecules in the construction of supramolecular architecture in the field of chemistry (Damiano *et al.*, 2007). Pyridinium cations are good candidates for second-harmonic generation (SHG) materials because they possess large hyperpolarizabilities (β) irrespective of the short cutoff wavelength. Since pyridinium cations are ionic species, they possess an easy tunability into noncentrosymmetric structures by changing counter anions (Anwar *et al.*, 1997, 1999). The zinc halides substituted in pyridines lead to a variety of complexes involving zinc centers and were shown to be catalytically active for the coupling of carbon dioxide and epoxides to provide high molecular weight polycarbonates and cyclic carbonates (Darensbourg *et al.*, 2003). As a part of our interest, we report here the crystal structure of the title pyridinium dichlorozinc(II) complex.

In the title molecule (Fig. 1), the Zn^II^ atom is coordinated by a pair of pyridinium oxide group and terminal halide ions in a distorted tetrahedral arrangement. The organic ligand exists in a zwitterionic structure, involving a conjugated pyridinium fragment. The C atoms of methyl substituents at C2, C6, C12 and C16 lie in the plane of the corresponding pyridinium rings, which are evident from the C9—C2—N1—C6 [176.99 (16)°], C10—C6—N1—C2 [-175.51 (17)°], C19—C12—N11—C16 [178.46 (18)°] and C20—C16—N11—C12 [-178.4 (2)°] torsion angles. The C—C bond of the ethyl groups attached at N1 and N11 are approximately perpendicular to the attached pyridinium ring, which can be seen from the C8—C7—N1—C2 [93.7 (2)°] and C18—C17—N11—C12 [90.0 (2)°] torsion angles. The sum of the bond angles around the protonated nitrogen atoms N1 [360.0°] and N11 [359.99°] of both the pyridinium rings is in accordance with *sp^2^* character. Due to protonation of N1 and N11 atoms of the pyridinium rings, the C2—N1—C6 and C12—N11—C16 angles are widened in comparison with the literature value (Mootz & Wusson, 1981). The pyridinium rings are planar and oriented each other at an angle of 67.79 (8)°.

The packing of the molecules in the unit cell is promoted by the existence of weak C—H···O, C—H···Cl and π···π types of intermolecular interactions. The C8—H8A···O1 interaction leads to the formation of a centrosymmetric R_2_^2^(16) dimer (Bernstein *et al.*, 1995). The Cl1 atom acts as an acceptor in a linear fashion for the methyl group hydrogen from the neighbouring molecule [Fig. 2 and Table 2]. The C13—H13···Cl2 intermolecular interaction also contributes to the crystal packing, which form zigzag chains along the *c* axis. The crystal structure is further augmented by π···π interaction between adjacent pyridinium rings [Cg1(x, y, z)···Cg1(-x, y, 1/2-z) = 3.669 (1) Å; where Cg1 is the centroid of the (N1-C6) ring, Fig.2].

### Experimental

1-Ethyl-2,6-dimethyl-4(1H)pyridinone trihydrate (EDMP.3H_2_O) was synthesized according to the reported method (Garratt, 1963). The title complex was prepared by the reaction of ZnCl_2_ with EDMP.3H_2_O in a 1:2 molar ratio in aqueous medium. Single crystals were harvested after a typical growth period of 15 days from a saturated aqueous solution at 303 K by slow evaporation of the solvent.

### Refinement

H atoms were positioned geometrically (C-H = 0.93-0.97 Å) and allowed to ride on their parent atoms, with U_iso_(H) = 1.5U_eq_(C) for methyl H and 1.2U_eq_(C) for other H atoms.

### Crystal data

**Table:**

[ZnCl_2_(C_9_H_13_NO)_2_]	*F*(000) = 1824
*M**_r_* = 438.68	*D*_x_ = 1.427 Mg m^−^^3^
Monoclinic, *C*2/*c*	Mo *K*α radiation, λ = 0.71073 Å
Hall symbol: -C 2yc	Cell parameters from 5069 reflections
*a* = 30.365 (2) Å	θ = 1.3–28.3°
*b* = 8.5366 (6) Å	µ = 1.48 mm^−^^1^
*c* = 15.7982 (12) Å	*T* = 293 K
β = 94.281 (4)°	Block, yellow
*V* = 4083.7 (5) Å^3^	0.25 × 0.25 × 0.23 mm
*Z* = 8	

### Data collection

**Table:**

Bruker SMART APEXII area-detector diffractometer	5069 independent reflections
Radiation source: fine-focus sealed tube	4248 reflections with *I* > 2σ(*I*)
graphite	*R*_int_ = 0.042
ω and φ scans	θ_max_ = 28.3°, θ_min_ = 1.3°
Absorption correction: multi-scan (*SADABS*, Bruker, 2008)	*h* = −40→40
*T*_min_ = 0.709, *T*_max_ = 0.727	*k* = −11→10
19140 measured reflections	*l* = −21→20

### Refinement

**Table:**

Refinement on *F*^2^	Secondary atom site location: difference Fourier map
Least-squares matrix: full	Hydrogen site location: inferred from neighbouring sites
*R*[*F*^2^ > 2σ(*F*^2^)] = 0.031	H-atom parameters constrained
*wR*(*F*^2^) = 0.084	*w* = 1/[σ^2^(*F*_o_^2^) + (0.0343*P*)^2^ + 3.3394*P*] where *P* = (*F*_o_^2^ + 2*F*_c_^2^)/3
*S* = 0.99	(Δ/σ)_max_ = 0.001
5069 reflections	Δρ_max_ = 0.61 e Å^−^^3^
233 parameters	Δρ_min_ = −0.56 e Å^−^^3^
0 restraints	Extinction correction: *SHELXL97*, Fc^*^=kFc[1+0.001xFc^2^λ^3^/sin(2θ)]^-1/4^
Primary atom site location: structure-invariant direct methods	Extinction coefficient: 0.00082 (11)

### Special details

**Table:**

Geometry. All esds (except the esd in the dihedral angle between two l.s. planes) are estimated using the full covariance matrix. The cell esds are taken into account individually in the estimation of esds in distances, angles and torsion angles; correlations between esds in cell parameters are only used when they are defined by crystal symmetry. An approximate (isotropic) treatment of cell esds is used for estimating esds involving l.s. planes.
Refinement. Refinement of F^2^ against ALL reflections. The weighted R-factor wR and goodness of fit S are based on F^2^, conventional R-factors R are based on F, with F set to zero for negative F^2^. The threshold expression of F^2^ > 2sigma(F^2^) is used only for calculating R-factors(gt) etc. and is not relevant to the choice of reflections for refinement. R-factors based on F^2^ are statistically about twice as large as those based on F, and R- factors based on ALL data will be even larger.

### Fractional atomic coordinates and isotropic or equivalent isotropic displacement parameters (Å^2^)

**Table:**

	*x*	*y*	*z*	*U*_iso_*/*U*_eq_	
C2	−0.01906 (5)	1.0155 (2)	0.10794 (11)	0.0359 (4)	
C3	0.01997 (6)	0.9693 (2)	0.14837 (11)	0.0374 (4)	
H3	0.0255	0.8629	0.1558	0.045*	
C4	0.05235 (5)	1.0787 (2)	0.17944 (11)	0.0364 (4)	
C5	0.04059 (5)	1.2375 (2)	0.16743 (12)	0.0383 (4)	
H5	0.0603	1.3145	0.1880	0.046*	
C6	0.00144 (6)	1.2821 (2)	0.12668 (11)	0.0377 (4)	
C7	−0.07005 (6)	1.2200 (3)	0.04757 (12)	0.0466 (4)	
H7A	−0.0797	1.1375	0.0082	0.056*	
H7B	−0.0646	1.3131	0.0147	0.056*	
C8	−0.10658 (6)	1.2540 (3)	0.10526 (14)	0.0568 (5)	
H8A	−0.1111	1.1639	0.1399	0.085*	
H8B	−0.1334	1.2777	0.0715	0.085*	
H8C	−0.0984	1.3419	0.1409	0.085*	
C9	−0.05273 (7)	0.8962 (3)	0.07675 (14)	0.0507 (5)	
H9A	−0.0810	0.9241	0.0956	0.076*	
H9B	−0.0442	0.7949	0.0988	0.076*	
H9C	−0.0545	0.8931	0.0159	0.076*	
C10	−0.00998 (7)	1.4522 (2)	0.11818 (15)	0.0554 (5)	
H10A	−0.0341	1.4755	0.1520	0.083*	
H10B	−0.0183	1.4759	0.0598	0.083*	
H10C	0.0152	1.5142	0.1374	0.083*	
C12	0.26273 (6)	0.9691 (2)	0.45067 (12)	0.0416 (4)	
C13	0.21875 (6)	0.9588 (2)	0.42739 (12)	0.0419 (4)	
H13	0.1987	0.9930	0.4650	0.050*	
C14	0.20270 (6)	0.8981 (2)	0.34832 (11)	0.0391 (4)	
C15	0.23516 (6)	0.8424 (2)	0.29655 (13)	0.0459 (4)	
H15	0.2263	0.7964	0.2447	0.055*	
C16	0.27915 (6)	0.8540 (2)	0.32029 (13)	0.0434 (4)	
C17	0.34104 (6)	0.9425 (2)	0.41988 (14)	0.0468 (4)	
H17A	0.3451	1.0363	0.4542	0.056*	
H17B	0.3561	0.9581	0.3686	0.056*	
C18	0.36164 (8)	0.8059 (3)	0.46811 (18)	0.0676 (7)	
H18A	0.3477	0.7925	0.5202	0.101*	
H18B	0.3926	0.8254	0.4805	0.101*	
H18C	0.3578	0.7127	0.4344	0.101*	
C19	0.27833 (7)	1.0377 (3)	0.53495 (15)	0.0652 (7)	
H19A	0.2934	1.1346	0.5264	0.098*	
H19B	0.2981	0.9657	0.5649	0.098*	
H19C	0.2534	1.0567	0.5676	0.098*	
C20	0.31287 (8)	0.7970 (4)	0.26254 (16)	0.0698 (7)	
H20A	0.2981	0.7509	0.2127	0.105*	
H20B	0.3315	0.7201	0.2916	0.105*	
H20C	0.3306	0.8836	0.2463	0.105*	
Cl1	0.08868 (2)	0.63005 (6)	0.23807 (5)	0.06526 (17)	
Cl2	0.163428 (18)	0.86170 (7)	0.10513 (3)	0.05362 (14)	
N1	−0.02813 (5)	1.17136 (17)	0.09508 (9)	0.0364 (3)	
N11	0.29302 (5)	0.92018 (19)	0.39652 (10)	0.0401 (3)	
O1	0.08947 (4)	1.04133 (16)	0.21754 (10)	0.0509 (3)	
O2	0.16095 (4)	0.89431 (19)	0.32761 (9)	0.0514 (3)	
Zn1	0.126091 (6)	0.85132 (2)	0.221676 (13)	0.03522 (8)	

### Atomic displacement parameters (Å^2^)

**Table:**

	*U*^11^	*U*^22^	*U*^33^	*U*^12^	*U*^13^	*U*^23^
C2	0.0331 (8)	0.0397 (9)	0.0349 (9)	−0.0027 (7)	0.0028 (6)	−0.0047 (7)
C3	0.0367 (8)	0.0331 (8)	0.0420 (10)	0.0009 (7)	0.0001 (7)	−0.0038 (7)
C4	0.0311 (8)	0.0414 (9)	0.0365 (9)	0.0019 (7)	0.0006 (6)	−0.0082 (7)
C5	0.0337 (8)	0.0372 (9)	0.0442 (10)	−0.0028 (7)	0.0040 (7)	−0.0086 (7)
C6	0.0366 (8)	0.0377 (9)	0.0395 (9)	0.0019 (7)	0.0082 (7)	−0.0019 (7)
C7	0.0382 (9)	0.0612 (12)	0.0391 (10)	0.0070 (8)	−0.0048 (7)	0.0064 (9)
C8	0.0358 (10)	0.0771 (15)	0.0569 (13)	0.0145 (10)	−0.0002 (9)	0.0079 (11)
C9	0.0406 (10)	0.0522 (11)	0.0583 (13)	−0.0106 (8)	−0.0028 (9)	−0.0086 (10)
C10	0.0540 (12)	0.0411 (11)	0.0712 (15)	0.0064 (9)	0.0054 (10)	0.0025 (10)
C12	0.0338 (8)	0.0507 (10)	0.0400 (10)	−0.0015 (7)	0.0008 (7)	−0.0080 (8)
C13	0.0310 (8)	0.0549 (11)	0.0399 (10)	0.0011 (7)	0.0034 (7)	−0.0087 (8)
C14	0.0317 (8)	0.0471 (10)	0.0380 (9)	0.0013 (7)	0.0003 (7)	−0.0002 (8)
C15	0.0391 (9)	0.0613 (12)	0.0366 (10)	0.0061 (8)	−0.0023 (7)	−0.0105 (8)
C16	0.0363 (9)	0.0532 (11)	0.0408 (10)	0.0074 (7)	0.0042 (7)	−0.0036 (8)
C17	0.0316 (8)	0.0511 (11)	0.0574 (12)	−0.0015 (8)	0.0010 (8)	0.0057 (9)
C18	0.0505 (13)	0.0731 (15)	0.0773 (17)	0.0136 (11)	−0.0076 (11)	0.0157 (13)
C19	0.0414 (11)	0.0977 (19)	0.0556 (13)	−0.0049 (11)	−0.0026 (9)	−0.0305 (13)
C20	0.0456 (12)	0.106 (2)	0.0580 (14)	0.0179 (12)	0.0083 (10)	−0.0213 (14)
Cl1	0.0609 (3)	0.0442 (3)	0.0936 (5)	−0.0149 (2)	0.0257 (3)	−0.0035 (3)
Cl2	0.0553 (3)	0.0670 (3)	0.0395 (3)	−0.0098 (2)	0.0095 (2)	0.0006 (2)
N1	0.0302 (7)	0.0440 (8)	0.0349 (8)	0.0032 (6)	0.0014 (5)	−0.0004 (6)
N11	0.0280 (7)	0.0478 (9)	0.0439 (8)	0.0019 (6)	−0.0009 (6)	−0.0009 (7)
O1	0.0375 (7)	0.0462 (7)	0.0660 (9)	0.0066 (6)	−0.0154 (6)	−0.0122 (7)
O2	0.0296 (6)	0.0840 (10)	0.0399 (7)	0.0028 (6)	−0.0032 (5)	−0.0090 (7)
Zn1	0.02879 (11)	0.03822 (13)	0.03829 (13)	−0.00233 (7)	0.00007 (8)	−0.00281 (8)

### Geometric parameters (Å, °)

**Table:**

C2—C3	1.362 (2)	C12—C19	1.498 (3)
C2—N1	1.371 (2)	C13—C14	1.406 (3)
C2—C9	1.500 (2)	C13—H13	0.93
C3—C4	1.416 (2)	C14—O2	1.286 (2)
C3—H3	0.93	C14—C15	1.409 (3)
C4—O1	1.278 (2)	C15—C16	1.364 (3)
C4—C5	1.412 (3)	C15—H15	0.93
C5—C6	1.363 (2)	C16—N11	1.368 (2)
C5—H5	0.93	C16—C20	1.502 (3)
C6—N1	1.372 (2)	C17—N11	1.490 (2)
C6—C10	1.496 (3)	C17—C18	1.503 (3)
C7—N1	1.488 (2)	C17—H17A	0.97
C7—C8	1.515 (3)	C17—H17B	0.97
C7—H7A	0.97	C18—H18A	0.96
C7—H7B	0.97	C18—H18B	0.96
C8—H8A	0.96	C18—H18C	0.96
C8—H8B	0.96	C19—H19A	0.96
C8—H8C	0.96	C19—H19B	0.96
C9—H9A	0.96	C19—H19C	0.96
C9—H9B	0.96	C20—H20A	0.96
C9—H9C	0.96	C20—H20B	0.96
C10—H10A	0.96	C20—H20C	0.96
C10—H10B	0.96	Cl1—Zn1	2.2292 (5)
C10—H10C	0.96	Cl2—Zn1	2.2349 (6)
C12—C13	1.361 (2)	O1—Zn1	1.9649 (13)
C12—N11	1.367 (2)	O2—Zn1	1.9472 (13)
			
C3—C2—N1	120.57 (15)	O2—C14—C15	124.24 (17)
C3—C2—C9	120.36 (17)	C13—C14—C15	115.41 (16)
N1—C2—C9	119.07 (16)	C16—C15—C14	121.93 (18)
C2—C3—C4	121.94 (16)	C16—C15—H15	119.0
C2—C3—H3	119.0	C14—C15—H15	119.0
C4—C3—H3	119.0	C15—C16—N11	120.19 (17)
O1—C4—C5	120.54 (16)	C15—C16—C20	120.50 (19)
O1—C4—C3	124.33 (17)	N11—C16—C20	119.30 (17)
C5—C4—C3	115.12 (15)	N11—C17—C18	112.90 (17)
C6—C5—C4	122.31 (16)	N11—C17—H17A	109.0
C6—C5—H5	118.8	C18—C17—H17A	109.0
C4—C5—H5	118.8	N11—C17—H17B	109.0
C5—C6—N1	120.23 (16)	C18—C17—H17B	109.0
C5—C6—C10	120.08 (17)	H17A—C17—H17B	107.8
N1—C6—C10	119.67 (16)	C17—C18—H18A	109.5
N1—C7—C8	112.78 (16)	C17—C18—H18B	109.5
N1—C7—H7A	109.0	H18A—C18—H18B	109.5
C8—C7—H7A	109.0	C17—C18—H18C	109.5
N1—C7—H7B	109.0	H18A—C18—H18C	109.5
C8—C7—H7B	109.0	H18B—C18—H18C	109.5
H7A—C7—H7B	107.8	C12—C19—H19A	109.5
C7—C8—H8A	109.5	C12—C19—H19B	109.5
C7—C8—H8B	109.5	H19A—C19—H19B	109.5
H8A—C8—H8B	109.5	C12—C19—H19C	109.5
C7—C8—H8C	109.5	H19A—C19—H19C	109.5
H8A—C8—H8C	109.5	H19B—C19—H19C	109.5
H8B—C8—H8C	109.5	C16—C20—H20A	109.5
C2—C9—H9A	109.5	C16—C20—H20B	109.5
C2—C9—H9B	109.5	H20A—C20—H20B	109.5
H9A—C9—H9B	109.5	C16—C20—H20C	109.5
C2—C9—H9C	109.5	H20A—C20—H20C	109.5
H9A—C9—H9C	109.5	H20B—C20—H20C	109.5
H9B—C9—H9C	109.5	C2—N1—C6	119.74 (14)
C6—C10—H10A	109.5	C2—N1—C7	120.04 (15)
C6—C10—H10B	109.5	C6—N1—C7	120.21 (15)
H10A—C10—H10B	109.5	C12—N11—C16	119.99 (15)
C6—C10—H10C	109.5	C12—N11—C17	119.84 (15)
H10A—C10—H10C	109.5	C16—N11—C17	120.15 (16)
H10B—C10—H10C	109.5	C4—O1—Zn1	134.31 (12)
C13—C12—N11	120.20 (16)	C14—O2—Zn1	133.29 (12)
C13—C12—C19	120.27 (17)	O2—Zn1—O1	98.22 (6)
N11—C12—C19	119.51 (16)	O2—Zn1—Cl1	107.99 (5)
C12—C13—C14	122.15 (17)	O1—Zn1—Cl1	114.29 (5)
C12—C13—H13	118.9	O2—Zn1—Cl2	115.09 (4)
C14—C13—H13	118.9	O1—Zn1—Cl2	105.10 (5)
O2—C14—C13	120.34 (16)	Cl1—Zn1—Cl2	115.06 (2)
			
N1—C2—C3—C4	0.7 (3)	C10—C6—N1—C7	4.0 (2)
C9—C2—C3—C4	−179.34 (17)	C8—C7—N1—C2	93.7 (2)
C2—C3—C4—O1	−179.80 (18)	C8—C7—N1—C6	−85.9 (2)
C2—C3—C4—C5	1.7 (3)	C13—C12—N11—C16	−3.0 (3)
O1—C4—C5—C6	179.59 (18)	C19—C12—N11—C16	178.4 (2)
C3—C4—C5—C6	−1.8 (3)	C13—C12—N11—C17	175.60 (18)
C4—C5—C6—N1	−0.4 (3)	C19—C12—N11—C17	−3.0 (3)
C4—C5—C6—C10	178.01 (17)	C15—C16—N11—C12	2.7 (3)
N11—C12—C13—C14	0.2 (3)	C20—C16—N11—C12	−178.4 (2)
C19—C12—C13—C14	178.8 (2)	C15—C16—N11—C17	−175.92 (18)
C12—C13—C14—O2	−178.01 (19)	C20—C16—N11—C17	3.0 (3)
C12—C13—C14—C15	2.7 (3)	C18—C17—N11—C12	90.0 (2)
O2—C14—C15—C16	177.7 (2)	C18—C17—N11—C16	−91.4 (2)
C13—C14—C15—C16	−3.0 (3)	C5—C4—O1—Zn1	−160.08 (14)
C14—C15—C16—N11	0.4 (3)	C3—C4—O1—Zn1	21.5 (3)
C14—C15—C16—C20	−178.5 (2)	C13—C14—O2—Zn1	169.24 (15)
C3—C2—N1—C6	−3.1 (3)	C15—C14—O2—Zn1	−11.6 (3)
C9—C2—N1—C6	176.98 (17)	C14—O2—Zn1—O1	−127.09 (19)
C3—C2—N1—C7	177.37 (16)	C14—O2—Zn1—Cl1	113.97 (19)
C9—C2—N1—C7	−2.6 (2)	C14—O2—Zn1—Cl2	−16.1 (2)
C5—C6—N1—C2	2.9 (3)	C4—O1—Zn1—O2	−159.12 (19)
C10—C6—N1—C2	−175.51 (17)	C4—O1—Zn1—Cl1	−45.1 (2)
C5—C6—N1—C7	−177.53 (17)	C4—O1—Zn1—Cl2	82.02 (19)

### Hydrogen-bond geometry (Å, °)

**Table:**

*D*—H···*A*	*D*—H	H···*A*	*D*···*A*	*D*—H···*A*
C8—H8A···O1^i^	0.96	2.53	3.344 (3)	143
C10—H10A···Cl1^ii^	0.96	2.82	3.740 (2)	162
C10—H10C···Cl1^iii^	0.96	2.82	3.745 (2)	162
C13—H13···Cl2^iv^	0.93	2.82	3.709 (2)	161

Symmetry codes: (i) −*x*, *y*, −*z*+1/2; (ii) −*x*, *y*+1, −*z*+1/2; (iii) *x*, *y*+1, *z*; (iv) *x*, −*y*+2, *z*+1/2.
